# Natural Products Targeting Airway Inflammation and Mucus Hypersecretion: Molecular Mechanisms and Therapeutic Potential for Respiratory Health

**DOI:** 10.3390/nu18162599

**Published:** 2026-08-08

**Authors:** Sung-Gyu Lee, Jae-Ho Lee, Hyun Kang

**Affiliations:** 1 Department of Medical Laboratory Science, College of Health Science, Dankook University, Cheonan 31116, Chungcheongnam-do, Republic of Korea; sung-gyu@dankook.ac.kr (S.-G.L.); dlwogh1214@naver.com (J.-H.L.); 2 Marine Bio-Food and Drug Convergence Technology Center, Dankook University, Cheonan 31116, Chungcheongnam-do, Republic of Korea

**Keywords:** airway inflammation, mucus hypersecretion, natural products, phytochemicals, marine bioactive compounds, MUC5AC, oxidative stress, NF-κB, respiratory diseases, nutraceuticals

## Abstract

Chronic respiratory diseases, including asthma, chronic obstructive pulmonary disease (COPD), bronchiectasis, cystic fibrosis, and chronic bronchitis, are characterized by persistent airway inflammation and mucus hypersecretion, leading to airway remodeling and progressive pulmonary dysfunction. Although current therapies improve disease control, they often fail to adequately target the complex molecular mechanisms underlying chronic airway diseases and may cause adverse effects during long-term use. Natural products have therefore emerged as promising multitarget therapeutic agents because they simultaneously regulate oxidative stress, inflammatory signaling, epithelial dysfunction, and mucus production. Recent evidence demonstrates that marine-derived bioactive compounds and plant-derived phytochemicals modulate key signaling pathways, including nuclear factor-kappa B (NF-κB), mitogen-activated protein kinases (MAPKs), phosphatidylinositol 3-kinase/protein kinase B (PI3K/Akt), Janus kinase/signal transducer and activator of transcription (JAK/STAT), the NOD-like receptor family pyrin domain-containing 3 (NLRP3) inflammasome, and nuclear factor erythroid 2-related factor 2 (Nrf2), thereby suppressing airway inflammation, oxidative stress, goblet cell differentiation, and MUC5AC overexpression. Advances in nanoformulation, pulmonary drug delivery, multi-omics, artificial intelligence-assisted drug discovery, and network pharmacology are expected to accelerate clinical translation. Collectively, natural products represent promising candidates for the development of evidence-based functional foods, nutraceuticals, and novel therapeutic strategies for chronic respiratory diseases.

## 1. Introduction

Chronic respiratory diseases (CRDs), including asthma, chronic obstructive pulmonary disease (COPD), bronchiectasis, cystic fibrosis (CF), and chronic bronchitis, remain among the leading causes of morbidity and mortality worldwide [1,2]. The increasing prevalence of air pollution, cigarette smoking, occupational exposure, and population aging has markedly increased the incidence of chronic airway diseases, imposing substantial healthcare and socioeconomic burdens [1,3]. Environmental pollutants such as fine particulate matter (PM_2.5_), allergens, and respiratory pathogens continuously disrupt airway homeostasis, resulting in persistent inflammation, progressive airway remodeling, and impaired pulmonary function [3,4]. Consequently, the development of safe and effective therapeutic strategies remains an important clinical challenge [5].

Airway inflammation is a hallmark of most chronic respiratory diseases and is initiated by excessive activation of airway epithelial cells and immune cells following exposure to environmental insults [3,4,5,6]. Activated epithelial cells release multiple pro-inflammatory mediators, including interleukin (IL)-1β, IL-6, IL-8, IL-13, tumor necrosis factor-alpha (TNF-α), and thymic stromal lymphopoietin (TSLP), which recruit inflammatory cells such as neutrophils, eosinophils, macrophages, and lymphocytes into the airway [5,6,7,8]. These responses are mediated through several intracellular signaling pathways, particularly nuclear factor-kappa B (NF-κB), mitogen-activated protein kinases (MAPKs), phosphatidylinositol 3-kinase/protein kinase B (PI3K/Akt), Janus kinase/signal transducer and activator of transcription (JAK/STAT), and the NOD-like receptor family pyrin domain-containing 3 (NLRP3) inflammasome, ultimately leading to chronic inflammation, oxidative stress, epithelial injury, and airway remodeling [6,7,8,9,10,11,12].

Another characteristic feature of chronic respiratory diseases is mucus hypersecretion, which contributes directly to airflow obstruction, recurrent infection, impaired mucociliary clearance, and disease progression [13,14,15,16]. Airway mucus normally functions as an essential defense barrier by trapping inhaled particles and microorganisms; however, excessive mucus production disrupts normal airway physiology [13,14]. Goblet cell hyperplasia and overproduction of the gel-forming mucin mucin 5AC (MUC5AC) are particularly prominent in asthma and COPD [13,14,15,16,17,18,19]. MUC5AC expression is stimulated by inflammatory cytokines, oxidative stress, and epidermal growth factor receptor (EGFR)-dependent signaling through NF-κB, MAPKs, PI3K/Akt, extracellular signal-regulated kinase (ERK), signal transducer and activator of transcription 6 (STAT6), transient receptor potential vanilloid 1 (TRPV1)/nuclear factor of activated T cells (NFAT)/TSLP, and transmembrane member 16A (TMEM16A)-related pathways [13,14,15,16,17,18,19,20]. Therefore, simultaneous suppression of airway inflammation and mucus hypersecretion has emerged as a key therapeutic strategy for improving respiratory function [13,14,15,16,17,18,19,20].

Current treatment of chronic airway diseases primarily includes inhaled corticosteroids, β2-adrenergic receptor agonists, muscarinic antagonists, phosphodiesterase (PDE) inhibitors, leukotriene receptor antagonists, and biologics targeting type 2 (T2) inflammatory cytokines [21]. Although these therapies effectively reduce symptoms and acute exacerbations, they frequently cause adverse effects following long-term administration and often fail to adequately control airway remodeling or mucus hypersecretion [21,22,23]. Moreover, the heterogeneous nature of chronic respiratory diseases suggests that targeting a single signaling pathway may be insufficient for achieving sustained therapeutic efficacy [10,11,12,21,22,23].

Natural products have therefore attracted considerable attention as potential multi-target therapeutic agents for respiratory diseases [3,5,22,23,24,25,26,27,28]. Bioactive compounds derived from medicinal plants, marine organisms, fungi, and microorganisms exhibit diverse pharmacological activities, including antioxidant, anti-inflammatory, immunomodulatory, and mucoregulatory effects [3,8,22,23,24,25,26,27,28]. Numerous phytochemicals, marine polyphenols, carotenoids, polysaccharides, terpenoids, alkaloids, and bioactive peptides have been shown to suppress inflammatory cytokine production, inhibit oxidative stress, regulate NF-κB, MAPKs, PI3K/Akt, JAK/STAT, stimulator of interferon genes (STING)/hypoxia-inducible factor-1α (HIF-1α), TRPV1/NFAT/TSLP, and ferroptosis-related signaling, restore epithelial barrier integrity, and reduce MUC5AC overexpression in experimental models of respiratory diseases [3,5,6,7,8,9,10,11,12,16,17,18,19,20,21,22,23,24,25,26,27,28]. Owing to their ability to simultaneously regulate multiple pathological pathways, natural products represent attractive candidates for the prevention and treatment of chronic airway disorders [22,23,24,25,26,27,28].

Although accumulating evidence supports their therapeutic potential, current studies largely focus on individual compounds or specific signaling pathways, and a comprehensive understanding of the molecular mechanisms by which natural products regulate both airway inflammation and mucus hypersecretion remains lacking [22,23,24,25,26,27,28]. Although several recent reviews have described the anti-inflammatory or antioxidant effects of natural products in respiratory diseases, few have comprehensively integrated the molecular mechanisms underlying both airway inflammation and mucus hypersecretion or critically discussed their translational potential as functional foods and nutraceuticals.

This review specifically focuses on chronic respiratory diseases characterized by persistent airway inflammation and mucus hypersecretion, including asthma, chronic obstructive COPD, bronchiectasis, CF, and chronic bronchitis. These diseases share common pathological features, such as chronic epithelial inflammation, goblet cell hyperplasia, excessive mucin production, and dysregulated inflammatory signaling pathways, making them particularly suitable for a comparative discussion of the molecular mechanisms targeted by natural products. In contrast, acute respiratory infections (e.g., pneumonia) and interstitial lung diseases (e.g., pulmonary fibrosis) involve distinct pathological mechanisms, including acute pathogen-driven inflammation or progressive fibrotic remodeling, and were therefore considered beyond the scope of the present review [29,30].

Therefore, this review summarizes recent advances in natural products targeting respiratory diseases, focusing on their molecular mechanisms, therapeutic efficacy, and translational potential. By integrating evidence from marine- and plant-derived natural products and highlighting common molecular targets, current evidence levels, and future clinical applications, this review provides a comprehensive perspective that may facilitate the development of evidence-based nutritional and therapeutic strategies for chronic respiratory diseases. We discuss the regulation of inflammatory signaling pathways, oxidative stress, epithelial barrier dysfunction, and mucin production, while highlighting future perspectives for developing evidence-based natural therapeutics to improve respiratory health.

## 2. Literature Search Strategy

This narrative review was based on a literature search conducted using PubMed, Web of Science, Scopus, and Google Scholar. Articles published between January 2015 and June 2026 were primarily included, while earlier landmark studies were considered when necessary to provide important background information. The search was performed using combinations of the following keywords: “airway inflammation”, “mucus hypersecretion”, “MUC5AC”, “natural products”, “marine natural products”, “phytochemicals”, “oxidative stress”, “NF-κB”, “MAPK”, “Nrf2”, “ferroptosis”, “respiratory diseases”, “asthma”, and “COPD”. Original research articles, clinical studies, and relevant review articles published in English were included. Priority was given to recent peer-reviewed studies with clear mechanistic or translational relevance to airway inflammation and mucus hypersecretion, whereas studies with limited relevance to the scope of this review were excluded. Representative natural products and bioactive compounds were selected based on the strength and consistency of experimental evidence, mechanistic relevance to airway inflammation and mucus hypersecretion, translational potential, and frequency of investigation across independent studies. When available, clinical studies and randomized controlled trials were additionally included to provide clinical perspectives and evaluate the translational applicability of promising natural products.

## 3. Molecular Mechanisms of Airway Inflammation

**Figure 1:**
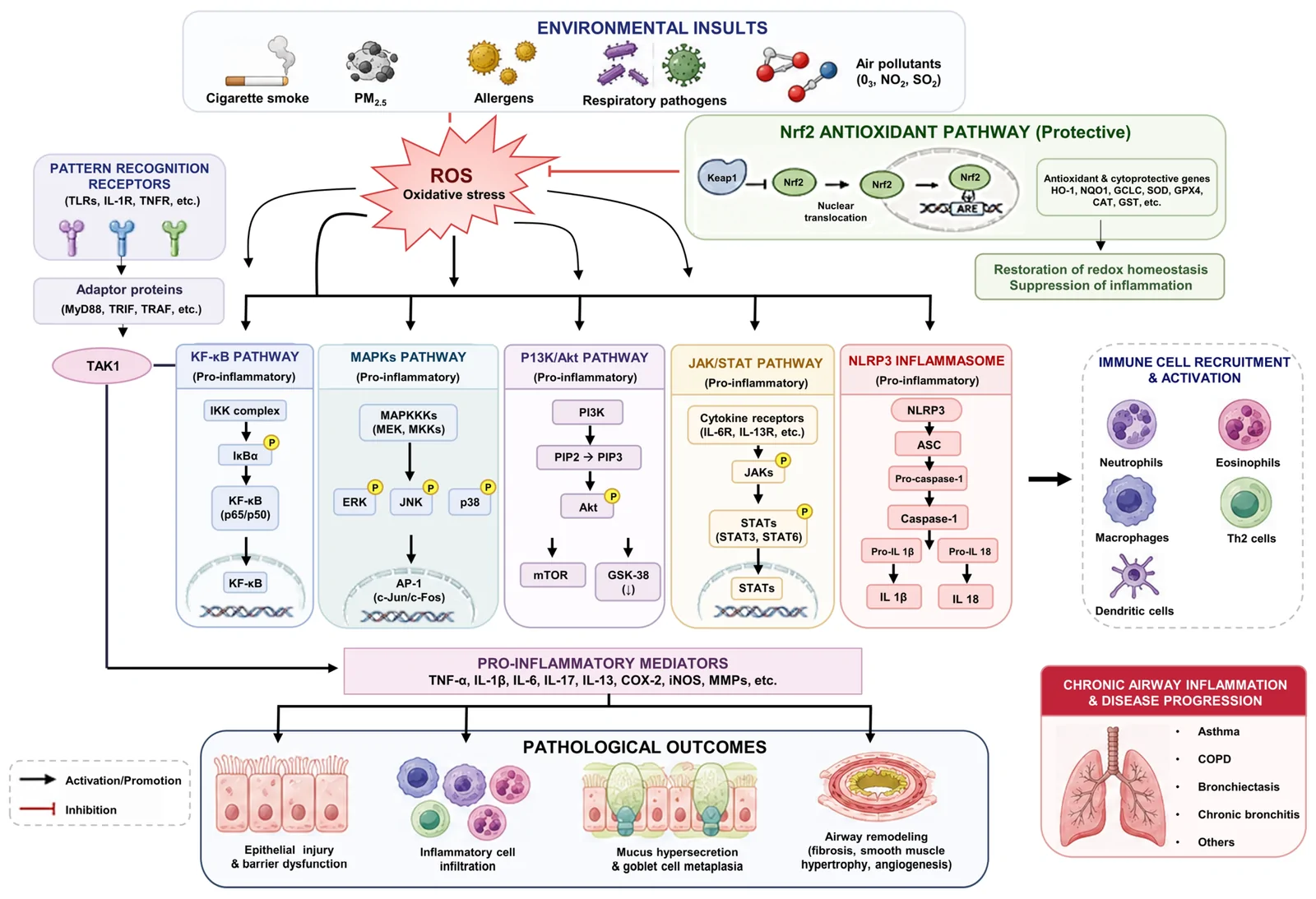
Molecular mechanisms underlying airway inflammation. Environmental insults induce oxidative stress and activate multiple inflammatory signaling pathways, including NF-κB, MAPKs, PI3K/Akt, JAK/STAT, and the NLRP3 inflammasome, leading to inflammatory mediator production, immune cell recruitment, epithelial injury, and airway remodeling. Activation of the Nrf2 antioxidant pathway counteracts oxidative stress and suppresses chronic airway inflammation.

Airway inflammation is a fundamental pathological process underlying the development and progression of chronic respiratory diseases, including asthma, COPD, bronchiectasis, and chronic bronchitis. Continuous exposure to environmental insults, such as PM, cigarette smoke, allergens, and respiratory pathogens, disrupts airway homeostasis and triggers a complex network of molecular events involving oxidative stress, epithelial barrier dysfunction, innate and adaptive immune activation, and metabolic reprogramming. These pathological responses are orchestrated through multiple interconnected signaling pathways, including NF-κB, MAPKs, PI3K/Akt, JAK/STAT, and the NLRP3 inflammasome, together with antioxidant defense systems regulated by nuclear factor erythroid 2-related factor 2 (Nrf2). These signaling pathways interact extensively to promote chronic airway inflammation, epithelial dysfunction, immune cell recruitment, mucus hypersecretion, and airway remodeling. Current understanding of these mechanisms is based largely on in vitro and animal studies, while clinical evidence remains limited. Therefore, further clinical validation is needed to confirm their relevance in human airway diseases. The major molecular mechanisms involved in the initiation and progression of airway inflammation are summarized in Figure 1.

### 3.1. Oxidative Stress and Redox Homeostasis

Oxidative stress is one of the earliest and most fundamental events driving the initiation and progression of chronic airway inflammation. Continuous exposure to environmental insults, including PM_2.5_, cigarette smoke, allergens, and respiratory pathogens, markedly increases intracellular reactive oxygen species (ROS) production in airway epithelial cells and resident immune cells. Excessive ROS not only causes oxidative damage to lipids, proteins, and DNA but also amplifies inflammatory signaling, epithelial barrier dysfunction, mucus hypersecretion, and airway remodeling, thereby contributing to the pathogenesis of asthma and COPD [31,32,33,34,35].

The Kelch-like ECH-associated protein 1 (Keap1)–Nrf2 pathway represents the principal endogenous defense system against oxidative injury. Under physiological conditions, Nrf2 is sequestered by Keap1 in the cytoplasm and undergoes ubiquitin-mediated degradation. Oxidative stress disrupts the Keap1–Nrf2 complex, allowing Nrf2 to translocate into the nucleus, where it binds to antioxidant response elements (AREs) and induces the transcription of multiple cytoprotective genes, including heme oxygenase-1 (HO-1), NAD(P)H quinone oxidoreductase-1 (NQO1), glutamate-cysteine ligase catalytic subunit (GCLC), and superoxide dismutase (SOD), thereby restoring intracellular redox homeostasis [8,9,22,36,37].

Accumulating evidence indicates extensive crosstalk between Nrf2 and pro-inflammatory signaling pathways. Nrf2 activation suppresses ROS-mediated activation of NF-κB, leading to reduced expression of inflammatory cytokines such as TNF-α, IL-1β, and IL-6, whereas sustained NF-κB activation further aggravates oxidative stress, creating a vicious cycle of chronic airway inflammation [22,32]. Recent studies have further demonstrated that Nrf2 signaling negatively regulates lipid peroxidation and ferroptotic cell death by maintaining glutathione metabolism and glutathione peroxidase 4 (GPX4) activity. For example, gentisic acid significantly attenuated ovalbumin-induced airway inflammation by simultaneously activating the Nrf2/HO-1 pathway, suppressing NF-κB signaling, and inhibiting ferroptosis, whereas the herbal formulation Divya-Swasari-Kwath alleviated airway inflammation and remodeling through robust activation of Nrf2-dependent antioxidant defenses [8,9]. However, current evidence is derived mainly from in vitro and animal studies, while clinical validation remains limited. Accordingly, clinical investigation is needed to confirm the therapeutic potential of Nrf2-targeting strategies in chronic airway diseases. Collectively, these findings suggest that restoration of redox homeostasis through Nrf2-mediated antioxidant signaling represents a promising therapeutic strategy for chronic respiratory diseases.

### 3.2. Canonical Pro-Inflammatory Signaling Pathways

Following oxidative stress, multiple intracellular signaling cascades orchestrate the initiation and amplification of airway inflammation. Among these, the NF-κB, MAPK, PI3K/Akt, JAK/STAT, and NLRP3 inflammasome pathways constitute the core pro-inflammatory signaling network that regulates cytokine production, immune-cell recruitment, epithelial injury, and airway remodeling [38,39,40,41,42].

Activation of Toll-like receptors (TLRs), interleukin-1 receptor (IL-1R), and tumor necrosis factor receptor (TNFR) triggers transforming growth factor-β-activated kinase 1 (TAK1)-dependent activation of the NF-κB and MAPK signaling pathways, leading to the production of pro-inflammatory mediators and immune-cell activation [5,6,7,38,39].

The PI3K/Akt, JAK/STAT, and NLRP3 inflammasome pathways further amplify inflammatory responses by promoting cytokine production, immune-cell activation, and airway remodeling [4,40,41,42]. Recent studies have shown that many natural products simultaneously suppress several of these signaling pathways rather than targeting a single molecule. For example, methyl lucidone inhibits TAK1 phosphorylation, thereby attenuating both NF-κB and MAPK activation in airway epithelial cells, whereas gentiopicroside suppresses SIRT1/NF-κB signaling and Epimedin C modulates noncanonical NF-κB and MAPK pathways, highlighting the multitarget mode of action of natural compounds for chronic airway diseases [5,6,7].

Although NF-κB serves as a common inflammatory signaling hub across chronic respiratory diseases, its upstream activators and downstream pathological consequences differ according to disease-specific immunopathology. In allergic asthma, NF-κB activation is primarily initiated by allergen exposure and epithelial-derived cytokines, leading to Th2-skewed immune responses, eosinophilic inflammation, goblet cell hyperplasia, and excessive MUC5AC production. In contrast, COPD is predominantly driven by cigarette smoke, air pollutants, and oxidative stress, resulting in persistent NF-κB activation associated with neutrophilic inflammation, macrophage recruitment, protease release, and progressive airway remodeling. Likewise, cystic fibrosis and bronchiectasis are characterized by chronic bacterial infection and sustained neutrophilic inflammation, in which prolonged NF-κB activation contributes to impaired resolution of inflammation and progressive tissue damage. These similarities and differences suggest that, although NF-κB represents a shared therapeutic target, disease-specific inflammatory mechanisms should be considered when developing natural product-based interventions for chronic respiratory diseases [38,39,40,41,42].

However, these findings are based mainly on preclinical studies, and clinical evidence remains limited. Further clinical studies are needed to confirm the therapeutic relevance of multitarget modulation in chronic airway diseases.

### 3.3. Airway Epithelial Signaling and Mucosal Immunity

The airway epithelium serves as the first line of defense against inhaled environmental insults and functions as an active immunological barrier that orchestrates innate and adaptive immune responses. Upon exposure to allergens, particulate matter, cigarette smoke, or respiratory pathogens, epithelial cells release alarmins, including TSLP, IL-25, and IL-33, which activate dendritic cells and group 2 innate lymphoid cells (ILC2s), thereby initiating type 2 inflammation and eosinophilic airway responses [16,17,43,44,45]. Among these mediators, TSLP has emerged as a central upstream regulator linking epithelial injury to allergic airway inflammation and mucus hypersecretion.

Transient receptor potential (TRP) channels have recently been recognized as important sensors of environmental stimuli in airway epithelial cells. Activation of TRPV1 promotes intracellular Ca^2+^ influx and NFAT activation, leading to increased TSLP production and amplification of type 2 immune responses [16,17]. Similarly, transient receptor potential ankyrin 1 (TRPA1) regulates epithelial inflammation and bronchoconstriction, whereas inhibition of the calcium-activated chloride channel TMEM16A effectively suppresses mucin secretion, highlighting the close relationship between epithelial signaling and mucus regulation [18,20,46].

Recent findings suggest that epithelial–immune cell crosstalk critically determines disease progression. DJ-1 promotes interactions between airway progenitor cells and eosinophils, thereby exacerbating allergic inflammation, while maintenance of epithelial barrier integrity through tight junction proteins attenuates inflammatory responses [19,20]. Notably, several natural products, including Quzhou Aurantii Fructus extract and Magnolia officinalis extract, alleviate airway inflammation by targeting the TRPV1/NFAT/TSLP signaling axis, emphasizing airway epithelial signaling as a key therapeutic target for chronic respiratory diseases [16,17]. However, current evidence is derived primarily from experimental studies, and clinical validation of these epithelial-targeting strategies remains limited.

### 3.4. Immunometabolic Regulation of Airway Inflammation

Recent studies indicate that immunometabolic reprogramming plays a pivotal role in the initiation and persistence of airway inflammation by integrating cellular metabolism with immune signaling. Upon exposure to allergens, particulate matter, or microbial components, airway epithelial cells and immune cells undergo metabolic remodeling characterized by enhanced glycolysis, mitochondrial dysfunction, and altered lipid metabolism, thereby supporting inflammatory activation and cytokine production [10,11,40,47,48,49]. HIF-1α is a central regulator of this process, promoting glycolytic metabolism and amplifying the production of pro-inflammatory mediators, including IL-1β and TNF-α [11,50]. In severe neutrophilic asthma, SIRT6-mediated metabolic reprogramming enhances lactate dehydrogenase A (LDHA)-dependent glycolysis, resulting in histone lactylation and increased expression of neutrophil-recruiting chemokines [10]. Moreover, activation of the STING pathway further potentiates HIF-1α-driven glycolysis and inflammatory responses [11,51]. Recent findings further suggest that mitochondrial metabolites such as succinate function as immunoregulatory signals that stabilize HIF-1α and activate inflammatory macrophages [52,53]. Importantly, several natural products have been shown to attenuate airway inflammation by modulating these immunometabolic pathways. For example, Qufeng Xuanbi formula suppresses the STING/HIF-1α/glycolysis axis, highlighting immunometabolism as a potential therapeutic target for chronic respiratory diseases [11]. However, current evidence is based mainly on experimental studies, and clinical validation of immunometabolic-targeting strategies remains limited.

### 3.5. Ferroptosis as an Emerging Driver of Airway Inflammation

Ferroptosis has recently emerged as a distinct form of regulated cell death characterized by iron-dependent lipid peroxidation and overwhelming oxidative membrane damage, contributing to the pathogenesis of chronic respiratory diseases [12,31,32,33,34,35,36,37,54]. Unlike apoptosis or necroptosis, ferroptosis is initiated by excessive accumulation of ROS, intracellular ferrous iron (Fe^2+^), and phospholipid hydroperoxides following impairment of the glutathione (GSH)/GPX4 antioxidant system [54,55,56]. Accumulating evidence suggests that ferroptotic airway epithelial cells release damage-associated molecular patterns (DAMPs), which amplify inflammatory signaling, recruit immune cells, and exacerbate epithelial barrier dysfunction and airway remodeling [12,22,57]. Recent findings indicate that ferroptosis interacts extensively with the NRF2/HO-1 antioxidant pathway, inflammatory NF-κB signaling, and mitochondrial dysfunction, establishing ferroptosis as a central mediator linking oxidative stress to chronic airway inflammation [9,12,35,36,37,58]. Importantly, several natural products suppress airway inflammation by inhibiting ferroptosis. Gentisic acid restored NRF2/HO-1 signaling and attenuated lipid peroxidation in ovalbumin-induced asthma, whereas linarin protected airway epithelial cells through ALDH2-dependent inhibition of ferroptosis [9,22]. Likewise, Quzhou Aurantii Fructus extract alleviated cough-variant asthma by simultaneously suppressing TRPV1/Ca^2+^/NFAT/TSLP signaling and ferroptosis, highlighting ferroptosis modulation as a potential therapeutic approach for respiratory diseases [16]. However, current evidence is derived primarily from experimental studies, and clinical validation of ferroptosis-targeting strategies remains limited.

## 4. Molecular Mechanisms of Airway Mucus Hypersecretion

**Figure 2:**
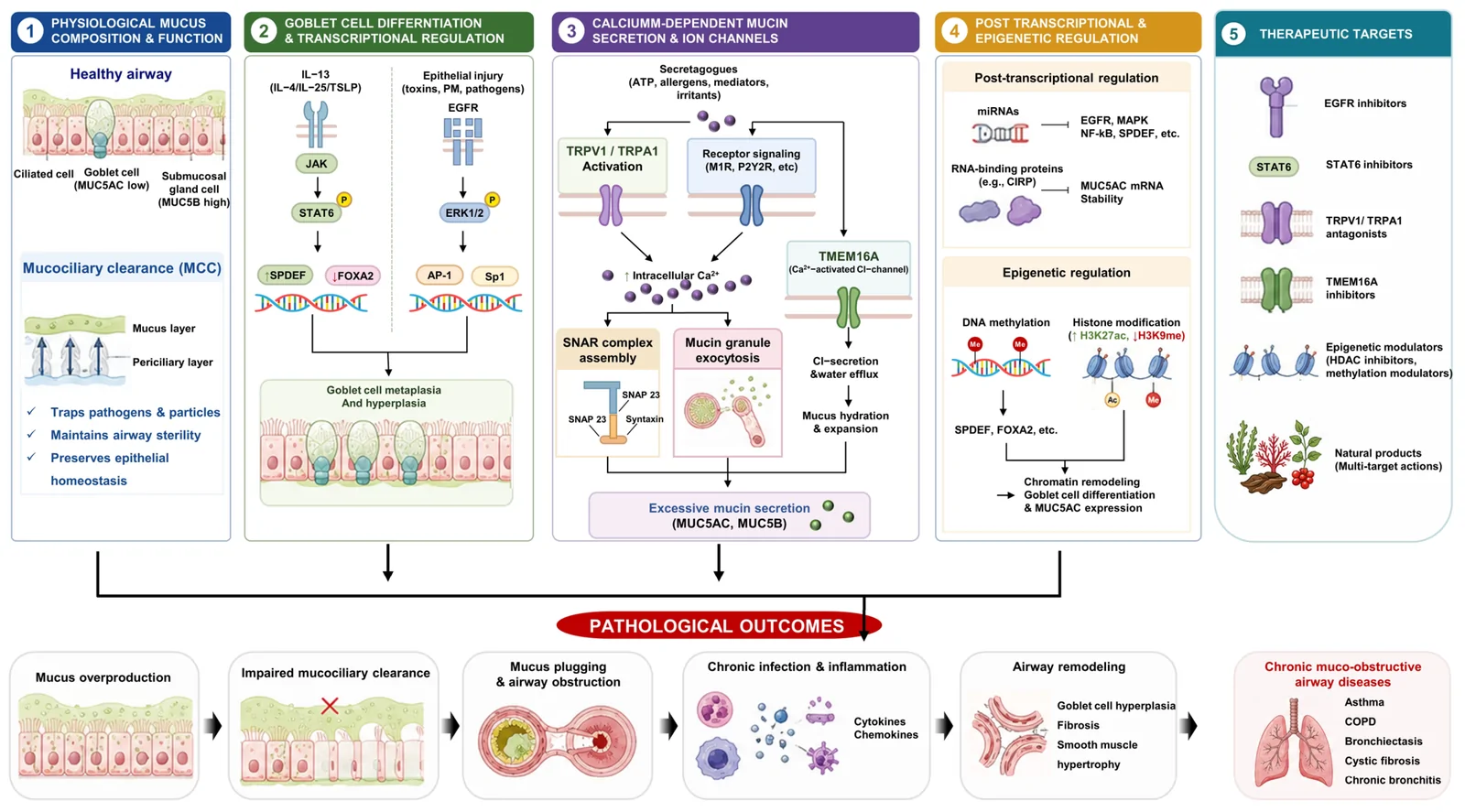
Molecular mechanisms underlying airway mucus hypersecretion. Environmental stimuli activate IL-13/STAT6/SPDEF, EGFR/MAPK, calcium-dependent ion channel signaling (TRPV1, TRPA1, and TMEM16A), and post-transcriptional and epigenetic regulatory mechanisms, resulting in goblet cell differentiation, MUC5AC overproduction, mucin secretion, and airway mucus hypersecretion.

Airway mucus hypersecretion is a characteristic pathological feature of chronic respiratory diseases, including asthma, COPD, bronchiectasis, cystic fibrosis, and chronic bronchitis. Under physiological conditions, mucus plays an essential role in mucociliary clearance by trapping inhaled pathogens, allergens, PM, and environmental pollutants. However, persistent inflammatory stimulation disrupts mucus homeostasis through coordinated alterations in mucin synthesis, goblet cell differentiation, calcium-dependent exocytosis, ion-channel activation, and post-transcriptional and epigenetic regulation. These processes collectively promote excessive MUC5AC expression, abnormal mucus secretion, impaired mucociliary clearance, and airway obstruction, thereby contributing to disease progression and recurrent airway injury. Recent studies have shown that multiple signaling pathways, including IL-13/STAT6/SAM-pointed domain-containing ETS transcription factor (SPDEF), EGFR/MAPK, TRPV1/TRPA1, TMEM16A-mediated calcium signaling, and epigenetic regulatory networks, interact to regulate both mucin production and secretion. Owing to the extensive crosstalk among these pathways, airway mucus hypersecretion has emerged as an important therapeutic target for chronic airway diseases. Current understanding of these mechanisms is based largely on experimental studies, while clinical validation remains limited. The major molecular mechanisms regulating airway mucus hypersecretion are summarized in Figure 2.

### 4.1. Physiological Functions and Composition of Airway Mucus

Airway mucus is a highly specialized viscoelastic secretion that constitutes the first line of defense against inhaled pathogens, allergens, particulate matter, and environmental pollutants. Together with coordinated ciliary beating, the mucus layer forms the mucociliary clearance (MCC) system, which continuously traps and removes foreign particles to maintain airway sterility and epithelial homeostasis [2,15,43,59,60]. The structural and rheological properties of airway mucus are primarily determined by two gel-forming mucins, MUC5AC and mucin 5B (MUC5B), which are synthesized by distinct epithelial cell populations [59,60,61]. Under physiological conditions, MUC5B, predominantly secreted by submucosal glands and secretory cells, is essential for effective mucus transport and host defense, whereas MUC5AC, mainly produced by surface goblet cells, is expressed at relatively low levels and is rapidly induced following epithelial stimulation [60,61,62]. Proper coordination between goblet cells, submucosal glands, airway surface liquid, and ciliary activity is therefore indispensable for maintaining efficient MCC [59,63]. Disruption of this delicate balance results in mucus dehydration, impaired mucus transport, and mucus plugging, which contribute to airflow obstruction, recurrent infection, and progressive airway dysfunction in chronic respiratory diseases, including asthma, COPD, bronchiectasis, and cystic fibrosis [15,59,61,62,63]. Accordingly, preservation of normal mucus composition and MCC has become an important therapeutic objective for preventing chronic airway disease progression.

### 4.2. Goblet Cell Differentiation and Transcriptional Regulation of MUC5AC

Goblet cell hyperplasia is a hallmark of chronic airway diseases and represents the primary cellular basis for excessive mucus production. IL-13 promotes goblet cell differentiation through the JAK/STAT6 pathway by increasing SPDEF expression and suppressing forkhead box A2 (FOXA2), thereby enhancing MUC5AC expression [42,44,47,64,65,66].

Airway epithelial injury induced by environmental pollutants, allergens, and microbial products also activates the EGFR/ERK pathway, further stimulating MUC5AC transcription [4,6,17,20,67,68]. Recent studies have shown that several natural products attenuate goblet cell differentiation by simultaneously suppressing the IL-13/STAT6/SPDEF axis and EGFR/ERK signaling, thereby restoring FOXA2 expression and reducing pathological MUC5AC overproduction [17,20,21,23]. However, these findings are based primarily on experimental studies, and their clinical relevance remains to be established.

### 4.3. Calcium-Dependent Mucin Secretion and Ion Channel Regulation

Although transcriptional regulation determines mucin synthesis, the final amount of mucus released into the airway lumen is regulated by Ca^2+^-dependent exocytosis of mucin granules. Secretory agonists, including ATP, allergens, inflammatory mediators, and environmental irritants, increase intracellular Ca^2+^ levels, triggering rapid mucin secretion through soluble N-ethylmaleimide-sensitive factor attachment protein receptor (SNARE)-mediated exocytosis [63,69,70].

As discussed above, TRPV1 and TRPA1 also contribute to calcium-dependent mucus secretion by promoting intracellular Ca^2+^ influx in airway epithelial cells, thereby linking epithelial immune signaling to mucin release [16,17,20,71,72]. Increased intracellular Ca^2+^ also activates TMEM16A, a calcium-activated chloride channel that regulates chloride and water secretion required for mucus hydration and efficient mucin granule expansion following exocytosis [18,46,73]. Recent findings indicate that pharmacological inhibition of TMEM16A markedly suppresses mucus secretion without directly affecting mucin gene transcription, highlighting TMEM16A as a key therapeutic target for mucus hypersecretion [18,73]. Several natural products have also been reported to alleviate mucus hypersecretion by modulating TRPV1- and TMEM16A-related signaling pathways [17,18,20]. Consequently, coordinated regulation of calcium signaling and ion-channel activity represents an important therapeutic strategy for controlling airway mucus hypersecretion.

### 4.4. Post-Transcriptional and Epigenetic Regulation of Mucin Production

Beyond transcriptional activation, airway mucin production is further refined by post-transcriptional and epigenetic mechanisms that determine mRNA stability, translation efficiency, and chromatin accessibility. Recent studies indicate that microRNAs (miRNAs) serve as critical post-transcriptional regulators of MUC5AC expression by targeting components of the EGFR, MAPK, NF-κB, and SPDEF signaling pathways. For example, miR-141, miR-146a, and several inflammation-responsive miRNAs modulate epithelial differentiation and mucus production, thereby influencing airway remodeling in asthma and chronic COPD [68,74,75,76]. In addition, RNA-binding proteins, including cold-inducible RNA-binding protein (CIRP), regulate MUC5AC mRNA stability, linking environmental stress to sustained mucus hypersecretion [74].

Epigenetic modifications further establish persistent mucin-producing phenotypes. DNA methylation within promoter regions of epithelial differentiation genes, particularly SPDEF, and dynamic histone modifications, including H3K27 acetylation and H3K9 methylation, alter chromatin accessibility and regulate goblet cell differentiation and mucin gene expression [75,76,77,78]. Histone deacetylase (HDAC)-dependent chromatin remodeling has also been recognized as an important mechanism controlling MUC5AC transcription, while short-chain fatty acids such as butyrate suppress mucin production by reversing histone acetylation at the MUC5AC promoter and restoring FOXA2 expression [78]. Taken together, post-transcriptional and epigenetic regulation provides long-term control of airway mucus production and represents a potential therapeutic target because these mechanisms integrate environmental exposure, inflammatory signaling, and epithelial plasticity into sustained mucus hypersecretion.

### 4.5. Therapeutic Targets for Airway Mucus Hypersecretion

The growing understanding of the molecular mechanisms governing airway mucus hypersecretion has facilitated the development of targeted therapeutic strategies aimed at restoring mucociliary homeostasis. Among these, inhibition of EGFR signaling remains one of the most extensively investigated approaches because EGFR activation directly induces MUC5AC transcription through the ERK/AP-1/Sp1 signaling cascade [4,6,17,67,68,79]. Although EGFR inhibitors effectively suppress mucin production in experimental models, their clinical application has been limited by adverse effects associated with systemic EGFR blockade.

TMEM16A has recently attracted considerable attention as a calcium-activated chloride channel that regulates airway surface hydration and mucin secretion. Pharmacological inhibition of TMEM16A significantly reduces mucus secretion and improves mucus clearance without directly interfering with epithelial viability, highlighting its potential as a valuable therapeutic target for chronic muco-obstructive diseases [18,46,73,80]. Likewise, antagonists targeting TRPV1 attenuate calcium influx, neurogenic inflammation, and mucus secretion induced by environmental irritants, while inhibition of STAT6 suppresses IL-13-driven goblet cell differentiation and MUC5AC overexpression, thereby interrupting one of the central pathways underlying type 2 airway inflammation [42,47,64,81].

Despite encouraging preclinical outcomes, therapies directed against single molecular targets often provide only partial clinical efficacy because airway mucus hypersecretion is regulated by highly interconnected signaling networks. Consequently, increasing interest has shifted toward multi-target therapeutic strategies, particularly bioactive natural products, which simultaneously modulate EGFR, STAT6, MAPKs, NF-κB, TMEM16A, oxidative stress, and epithelial barrier function. Numerous phytochemicals, marine-derived compounds, and microbial metabolites have demonstrated the ability to suppress both airway inflammation and mucus hypersecretion through coordinated regulation of multiple signaling pathways [3,5,6,7,8,9,10,11,12,13,14,15,16,17,18,19,20,21,22,23,24,25,68,78,82]. However, most of these findings are derived from preclinical studies, and further clinical studies are needed to validate their therapeutic efficacy. These multitarget mechanisms provide the rationale for the following section, which comprehensively summarizes natural products with potential therapeutic value for chronic respiratory diseases.

## 5. Natural Products Targeting Airway Inflammation and Mucus Hypersecretion

### 5.1. Marine-Derived Natural Products

Among marine-derived bioactive compounds, dieckol, a major phlorotannin isolated from *Ecklonia cava*, exhibits potent antioxidant, anti-inflammatory, and mucoregulatory activities. Owing to its multiple hydroxyl groups, dieckol effectively scavenges ROS and activates the Nrf2/HO-1 pathway while suppressing NF-κB, MAPKs, and PI3K/Akt signaling, thereby reducing the production of pro-inflammatory mediators [3,31,32,33,34,35,36,37,38,39,40,41,42,83]. Recent studies have shown that dieckol suppresses MUC5AC expression, attenuates goblet cell hyperplasia, and alleviates particulate matter-induced pulmonary inflammation through inhibition of MAPK signaling [3]. These multitarget properties support the potential application of dieckol in preventing chronic airway inflammation and mucus hypersecretion [83,84].

In addition to dieckol, other marine phlorotannins, including eckol and phlorofucofuroeckol A (PFF-A), have demonstrated considerable pharmacological activity against airway inflammation. Eckol exerts antioxidant and anti-inflammatory effects by attenuating NF-κB and MAPK signaling while preserving epithelial integrity and reducing oxidative injury [85,86]. More recently, PFF-A has emerged as a potential therapeutic candidate for particulate matter-induced respiratory inflammation. In fine dust-stimulated macrophages, PFF-A markedly suppressed IL-1β, TNF-α, and chemokine expression through inhibition of the NF-κB/MAPK pathways, while simultaneously reducing apoptosis-related gene expression and restoring cytoprotective signaling [86]. Furthermore, phlorotannin-rich Eisenia bicyclis extracts containing eckol and PFF-A alleviated PM_2.5_-induced chronic lung injury by activating Nrf2/HO-1 signaling and suppressing TLR4, cyclooxygenase-2 (COX-2), and transforming growth factor (TGF)-β/Smad pathways, highlighting their potential as multifunctional marine therapeutics for chronic respiratory diseases.

Marine carotenoids have also attracted considerable attention owing to their potent antioxidant and immunomodulatory activities. Fucoxanthin, a xanthophyll carotenoid abundant in brown seaweeds, has been shown to attenuate airway inflammation by suppressing oxidative stress and inhibiting NF-κB, MAPK, and PI3K/Akt signaling pathways, thereby reducing the production of pro-inflammatory cytokines and inflammatory mediators. Recent studies have shown that fucoxanthin alleviates airway remodeling, epithelial injury, and mucus hypersecretion while enhancing Nrf2/HO-1-mediated antioxidant defense, suggesting its potential application in asthma and COPD [83,84,87,88,89]. Similarly, astaxanthin, a marine ketocarotenoid predominantly produced by *Haematococcus pluvialis*, exhibits strong free radical-scavenging capacity and protects airway epithelial cells against particulate matter-induced oxidative damage. Astaxanthin suppresses inflammatory cytokine production through inhibition of NF-κB and MAPKs while restoring mitochondrial function and epithelial barrier integrity. Recent in vivo studies have shown that astaxanthin ameliorates pulmonary inflammation and oxidative injury by activating the Nrf2/HO-1 pathway and reducing inflammatory cell infiltration [83,84,89,90,91].

Among marine sterols, fucosterol has been recognized as another bioactive compound with broad anti-inflammatory properties. Fucosterol inhibits inflammatory mediator production by regulating NF-κB, MAPKs, and Nrf2 signaling and has recently been reported to alleviate airway inflammation and epithelial dysfunction in experimental respiratory disease models. In addition, molecular docking and network pharmacology analyses suggest that fucosterol interacts with multiple inflammatory targets, supporting its multitarget mode of action [3,83,84].

Marine-derived omega-3 polyunsaturated fatty acids (PUFAs), particularly eicosapentaenoic acid (EPA) and docosahexaenoic acid (DHA), together with their specialized pro-resolving mediators (SPMs), including resolvins, protectins, and maresins, have recently emerged as important regulators of airway inflammation [92,93]. Unlike conventional anti-inflammatory agents that primarily suppress inflammatory signaling, SPMs actively promote the resolution of inflammation by limiting neutrophil infiltration, enhancing macrophage-mediated clearance of inflammatory cells, and restoring tissue homeostasis [92,93]. Recent studies have demonstrated that omega-3-derived lipid mediators attenuate airway inflammation by regulating COX-2-derived lipid mediator pathways, suppressing NF-κB activation, reducing pro-inflammatory cytokine production, and promoting inflammation resolution [92,93,94]. In experimental models of asthma and COPD, omega-3-derived mediators reduced airway inflammation, goblet cell hyperplasia, oxidative stress, and airway remodeling, suggesting that marine lipid mediators complement other marine-derived bioactive compounds by targeting both inflammatory initiation and resolution pathways [94,95].

Taken together, marine-derived compounds, including phlorotannins, carotenoids, sterols, omega-3 polyunsaturated fatty acids, and specialized pro-resolving mediators, share common mechanisms involving suppression of NF-κB, MAPKs, PI3K/Akt, and EGFR signaling, activation of the Nrf2/HO-1 antioxidant pathway, inhibition of MUC5AC overproduction, modulation of COX-2-related lipid mediator pathways, and emerging regulation of ferroptosis. These multitarget activities highlight the unique therapeutic advantages of marine natural products and provide a mechanistic framework for the plant-derived bioactive compounds discussed in the following section. However, most available evidence is derived from in vitro and animal studies, and clinical studies evaluating the efficacy of marine-derived compounds in chronic airway diseases remain limited.

### 5.2. Plant-Derived Bioactive Compounds

Plant-derived phytochemicals represent the largest and most extensively studied class of natural products targeting airway inflammation and mucus hypersecretion. Although numerous compounds have demonstrated significant biological activities, most evidence has been obtained from experimental studies, and relatively few have progressed to clinical evaluation. These compounds include flavonoids, phenolic acids, stilbenes, terpenoids, anthraquinones, saponins, and glycosides, many of which exert pleiotropic effects on oxidative stress, epithelial activation, type 2 inflammation, and mucin production. Unlike single-target pharmacological inhibitors, plant phytochemicals often regulate multiple interconnected pathways, including NF-κB, MAPKs, EGFR, STAT6, Nrf2/HO-1, and MUC5AC/MUC5B signaling networks [5,6,7,8,9,16,17,20,21,22,23,24,25,26,27,28,82,96,97,98,99,100,101,102,103,104,105].

Flavonoids are particularly relevant to airway mucus regulation. Liquiritin, a flavonoid derived from licorice, has been shown to suppress both intracellular and secreted MUC5AC and MUC5B in NCI-H292 human airway epithelial cells, accompanied by reduced ERK and p38 MAPK phosphorylation [82]. Similarly, taxifolin inhibited PMA-induced MUC5AC mucin gene expression by preventing IκBα degradation and NF-κB p65 nuclear translocation, suggesting that flavonoids can directly modulate mucin transcription through inflammatory transcription factor blockade [96]. Luteolin, quercetin, and eriodictyol have also been reported to reduce mucin production and airway inflammatory responses by targeting NF-κB, EGFR, and MAPK-related pathways [97,98,99]. In particular, quercetin attenuated cigarette smoke-induced airway inflammation and mucus production by suppressing oxidative stress, EGFR phosphorylation, and NF-κB activation [97].

Phenolic acids and stilbenes further contribute to airway protection by regulating redox-sensitive inflammatory signaling. Resveratrol inhibited LPS-induced MUC5AC expression and airway inflammation through coordinated regulation of MAPK and Nrf2 pathways, highlighting the importance of antioxidant response restoration in mucus control [100]. Neochlorogenic acid improved allergic airway inflammation by suppressing type 2 immune responses and activating Nrf2/HO-1, thereby reducing oxidative stress and inflammatory cell infiltration [101]. These findings are consistent with previous studies showing that antioxidant phytochemicals can suppress cytokine-driven goblet cell responses and epithelial injury.

Several structurally distinct plant-derived compounds directly regulate MUC5AC transcription. Emodin, an anthraquinone found in rhubarb and buckthorn, inhibited EGF-induced MUC5AC expression by suppressing the EGFR-MAPK-Sp1 signaling axis in NCI-H292 cells [102]. Likewise, hederacoside C, a triterpenoid saponin from Hedera helix, reduced EGF-induced MUC5AC mucin gene expression through inhibition of the MAPK pathway [103]. Iristectorigenin A attenuated airway inflammation, goblet cell hyperplasia, and mucus hypersecretion in OVA-induced asthmatic mice by regulating type 2 immune responses and suppressing FOXA3 and NOTCH2 signaling [104]. More recently, Raphanus sativus root extracts significantly reduced PMA-induced mucus secretion and MUC5AC mRNA expression in NCI-H292 cells, supporting the value of edible plant materials as respiratory health-promoting functional ingredients [105].

Vitamins also represent an important class of naturally occurring bioactive compounds that contribute to respiratory health through antioxidant and immunomodulatory mechanisms [106]. Among these, vitamins E and D have been extensively investigated in experimental models of chronic airway diseases [107,108]. Vitamin E (α-tocopherol), a lipid-soluble antioxidant abundant in edible plant oils, nuts, and seeds, has been shown to attenuate cigarette smoke-induced oxidative stress, inflammatory cell infiltration, and pulmonary injury by suppressing NF-κB activation and restoring endogenous antioxidant defenses in preclinical COPD models [107]. Likewise, vitamin E reduced eosinophilic airway inflammation and airway hyperresponsiveness in experimental asthma by decreasing Th2-associated cytokine production and oxidative damage [108]. Vitamin D has also emerged as a promising regulator of airway immune homeostasis [106,109]. Recent studies demonstrated that vitamin D suppresses Th2-mediated airway inflammation, reduces goblet cell hyperplasia and MUC5AC expression, and improves epithelial barrier integrity through modulation of NF-κB, MAPK, and JAK/STAT signaling pathways [109]. Collectively, these findings suggest that vitamins complement other plant-derived phytochemicals by simultaneously regulating oxidative stress, airway inflammation, and mucus hypersecretion, thereby broadening the therapeutic potential of plant-derived natural products for respiratory health [106,107,108,109].

Taken together, plant-derived phytochemicals suppress airway inflammation and mucus hypersecretion through convergent regulation of oxidative stress, epithelial inflammatory signaling, goblet cell differentiation, and mucin gene expression. Their ability to simultaneously modulate NF-κB/MAPK, EGFR-Sp1, STAT6, Nrf2/HO-1, and MUC5AC/MUC5B pathways provides strong rationale for their development as nutraceuticals, functional foods, or adjunctive therapeutics for chronic respiratory diseases. Many of these phytochemicals, together with naturally occurring bioactive vitamins, are present in edible fruits, vegetables, nuts, seeds, and medicinal plants, supporting their application in functional foods and nutraceutical formulations. However, further studies are needed to optimize food matrices, formulation strategies, and effective dietary intake levels to maximize their bioavailability and health benefits. Nevertheless, most of the supporting evidence remains preclinical, and further well-designed clinical studies are required to confirm their efficacy and safety in humans. The representative marine- and plant-derived natural products discussed in this section, together with their principal molecular targets and pharmacological activities against airway inflammation and mucus hypersecretion, are summarized in Table 1.

## 6. Future Perspectives

Despite the remarkable progress in elucidating the molecular mechanisms underlying the anti-inflammatory and mucoregulatory activities of natural products, several critical challenges remain before their successful clinical translation for respiratory diseases. One of the major limitations is the poor oral bioavailability of many phytochemicals and marine-derived metabolites. Polyphenolic compounds such as dieckol, fucoxanthin, quercetin, and resveratrol exhibit low aqueous solubility, limited intestinal permeability, extensive first-pass metabolism, and rapid systemic elimination, resulting in insufficient concentrations at pulmonary target sites despite encouraging in vitro efficacy [83,84,110,111]. These pharmacokinetic limitations frequently result in low systemic exposure after oral administration, representing a major barrier to the clinical translation of otherwise promising natural products. Therefore, comprehensive pharmacokinetic (PK) and pharmacodynamic (PD) investigations are essential to establish absorption, tissue distribution, metabolism, elimination, optimal dosing regimens, and long-term safety profiles before these compounds can be translated into evidence-based therapeutics.

Successful clinical translation of natural products into therapeutic agents, functional foods, and nutraceuticals remains limited because of poor pharmacokinetic properties, variability in extract composition, and the lack of standardized formulations. In addition, insufficient dose optimization, limited high-quality clinical trials, and regulatory challenges continue to hinder their clinical application. Addressing these issues will be essential for successful clinical translation.

Beyond pharmacokinetic limitations, several practical and commercial barriers continue to delay the clinical translation of natural products [112,113]. Compared with conventional synthetic drugs, natural products often contain multiple bioactive constituents that simultaneously modulate numerous molecular targets [112]. Although this multitarget mode of action may provide therapeutic advantages for complex chronic diseases, it also complicates precise target validation, biomarker selection, and regulatory evaluation [112,113]. Furthermore, intellectual property protection for naturally occurring compounds is often limited, reducing commercial incentives for pharmaceutical investment and large-scale clinical development [112,113]. The substantial costs associated with standardized manufacturing, multicenter clinical trials, and regulatory approval further restrict industrial uptake, particularly for nutraceuticals and functional foods [112,113]. Addressing these scientific, regulatory, and commercial challenges through interdisciplinary collaboration among academia, industry, and regulatory agencies will be essential to accelerate the successful translation of natural products into evidence-based clinical applications.

Another important issue is the standardization and quality control of natural products. The chemical composition and biological activity of botanical and marine extracts are strongly influenced by species, geographic origin, harvesting season, cultivation conditions, extraction procedures, and purification methods. Consequently, substantial batch-to-batch variability frequently compromises reproducibility among preclinical studies and complicates regulatory approval. Future investigations should therefore emphasize standardized manufacturing processes, quantitative marker compounds, validated analytical platforms, and good manufacturing practice (GMP)-compliant production to ensure consistent efficacy, safety, and product quality [83,84].

Advanced nanoformulation technologies provide effective strategies to overcome these pharmacokinetic limitations. Nanoemulsions, liposomes, polymeric nanoparticles, solid lipid nanoparticles, cyclodextrin inclusion complexes, and polylactide-co-glycolide (PLGA)-based delivery systems have demonstrated considerable potential for enhancing the solubility, stability, controlled release, and tissue-specific accumulation of poorly bioavailable natural compounds. These approaches not only improve systemic exposure but also protect labile phytochemicals from degradation and enhance intracellular uptake, thereby increasing therapeutic efficacy while minimizing systemic toxicity [106,108,109].

**Figure 3:**
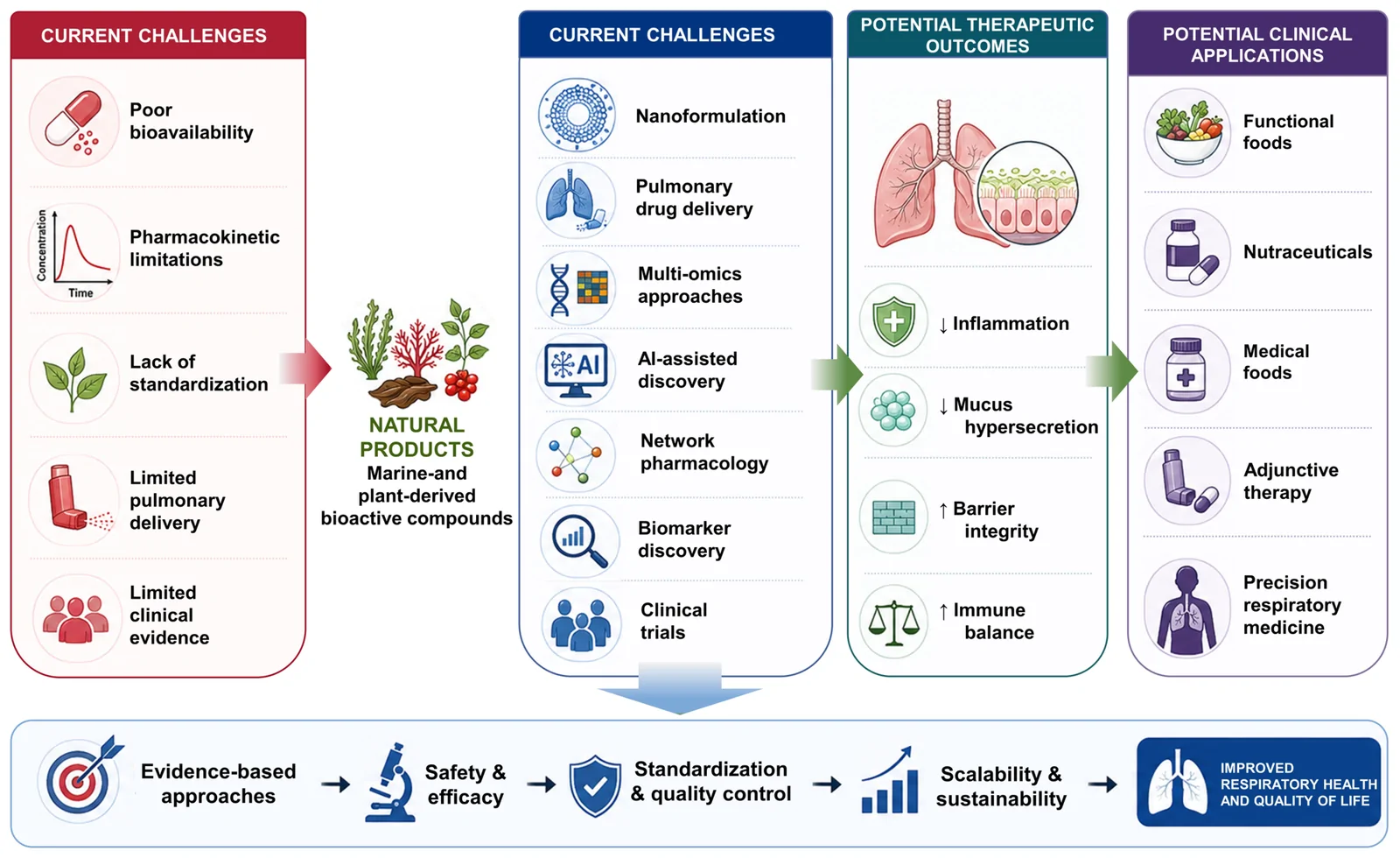
Future perspectives for the clinical translation of natural products targeting airway inflammation and mucus hypersecretion. Major translational challenges, emerging technologies, and future strategies for developing natural products into evidence-based interventions for chronic airway diseases. Advances in nanoformulation, pulmonary drug delivery, multi-omics, AI-assisted discovery, network pharmacology, biomarker-guided approaches, and clinical validation are expected to accelerate the development of functional foods, nutraceuticals, and precision respiratory therapeutics. These proposed applications are supported primarily by preclinical evidence and require further clinical validation.

For respiratory diseases, pulmonary drug delivery represents an especially attractive translational strategy because inhalation directly targets diseased airways while bypassing hepatic first-pass metabolism. Recent advances in inhalable nano and microparticle formulations have substantially improved pulmonary deposition efficiency, local drug retention, and controlled release within the respiratory tract. Recently developed nebulized formulations of natural products further demonstrate enhanced lung bioavailability and favorable safety profiles, highlighting the potential of inhalation-based delivery systems to maximize local pharmacological activity while reducing off-target adverse effects [110,114,115,116,117]. Overall, overcoming the current translational barriers will require the integration of advanced drug delivery technologies, systems biology, computational approaches, and rigorous clinical validation. Figure 3 summarizes the major challenges, emerging technologies, therapeutic outcomes, and future translational strategies for developing evidence-based natural product interventions for chronic airway diseases.

Recent advances in multi-omics technologies are expected to substantially accelerate the discovery and mechanistic validation of natural products for chronic airway diseases. Integrated analyses combining genomics, transcriptomics, proteomics, metabolomics, and lipidomics can comprehensively characterize disease-associated molecular networks and identify biomarkers predictive of therapeutic responses. Furthermore, emerging single-cell RNA sequencing and spatial transcriptomics provide unprecedented resolution for defining cell-type-specific signaling alterations within the airway epithelium, immune cell populations, and stromal microenvironment. These technologies will facilitate the identification of novel therapeutic targets and enable precision intervention strategies tailored to distinct inflammatory endotypes of asthma, COPD, and other chronic respiratory disorders [77,117].

The integration of artificial intelligence (AI) with systems biology is also transforming natural product research. AI-assisted virtual screening, machine learning-based structure–activity relationship (SAR) prediction, molecular docking, and molecular dynamics simulations considerably improve the efficiency of identifying bioactive compounds and optimizing lead candidates. When combined with network pharmacology, AI enables systematic prediction of multitarget interactions, signaling networks, and synergistic effects among complex phytochemical mixtures, thereby overcoming one of the major limitations of conventional single-target drug discovery [116,117]. Such computational approaches are particularly valuable for marine-derived polyphenols and botanical extracts that contain numerous structurally diverse constituents acting on interconnected inflammatory pathways.

Another limitation of the present review is that chronic respiratory diseases represent heterogeneous clinical entities with distinct immunopathological characteristics [118,119,120,121]. Although this review emphasizes the common molecular mechanisms underlying airway inflammation and mucus hypersecretion, disease-specific inflammatory endotypes and therapeutic responses were not comprehensively discussed [118,119,120,121]. For example, asthma comprises both type 2 and non-type 2 inflammatory endotypes, which differ substantially in cytokine profiles, immune-cell composition, and responsiveness to biological therapies [118,119]. Likewise, COPD is predominantly characterized by neutrophilic inflammation and oxidative stress, whereas cystic fibrosis and bronchiectasis are strongly associated with chronic bacterial infection, impaired mucociliary clearance, and persistent neutrophilic inflammation [120,121]. Consequently, although natural products frequently target shared signaling pathways such as NF-κB, MAPKs, PI3K/Akt, and Nrf2/HO-1, their therapeutic efficacy may vary according to disease-specific pathophysiological mechanisms [118,119,120,121]. Future studies should therefore incorporate disease-specific molecular signatures and inflammatory endotypes to facilitate precision nutrition and personalized therapeutic strategies for chronic respiratory diseases [118,119,120,121].

Despite the growing body of encouraging preclinical evidence, translation into clinical practice has been slow. Most natural products have been evaluated primarily in cell culture and animal models, whereas randomized, placebo-controlled clinical trials investigating respiratory outcomes remain scarce. Future studies should therefore emphasize standardized formulations, pharmacokinetic-guided dose optimization, long-term safety assessments, and well-designed multicenter clinical trials using validated biomarkers, pulmonary function tests, and patient-reported outcomes to establish robust clinical evidence [83,110,114,115].

Although most natural products remain at the preclinical stage, several clinical studies have evaluated selected natural products as adjunctive interventions for chronic respiratory diseases, particularly asthma and COPD, with encouraging but still preliminary findings [93,122,123,124]. Randomized clinical trials and observational studies have investigated herbal medicines, vitamin D supplementation, and selected phytochemicals, whereas the clinical evidence for omega-3 fatty acids remains comparatively limited and heterogeneous [93,122,123,124]. Some studies have reported improvements in respiratory symptoms, quality of life, inflammatory biomarkers, and pulmonary function; however, these findings should be interpreted with caution because of small sample sizes, heterogeneous formulations, variable dosing regimens, differences in study populations, and inconsistent clinical endpoints [93,122,123,124]. Therefore, future well-designed, multicenter, randomized controlled trials using standardized formulations, validated biomarkers, and clinically meaningful respiratory outcomes are essential to establish the efficacy, safety, and translational potential of natural product-based interventions for chronic respiratory diseases [93,122,123,124].

Finally, natural products possess considerable opportunities for development as functional foods, nutraceuticals, and adjunctive therapies for maintaining respiratory health. Unlike conventional anti-inflammatory drugs that generally target individual signaling pathways, many dietary phytochemicals and marine bioactives simultaneously modulate oxidative stress, inflammatory signaling, epithelial barrier integrity, mucus hypersecretion, immunometabolism, and ferroptosis. Future research should focus on developing standardized functional food and nutraceutical formulations with improved stability, bioavailability, and consumer acceptability, while also establishing evidence-based dietary intake recommendations through well-designed clinical studies. Future interdisciplinary collaboration integrating nutritional science, pharmaceutical technology, computational biology, clinical medicine, and precision medicine approaches will facilitate the development of evidence-based functional ingredients tailored to distinct inflammatory endotypes and chronic respiratory diseases [83,84,114,117].

## 7. Conclusions

Airway inflammation and mucus hypersecretion are fundamental pathological features shared by numerous chronic respiratory diseases, including asthma, COPD, chronic bronchitis, cystic fibrosis, and bronchiectasis. Accumulating evidence suggests that these pathological processes are orchestrated through highly interconnected molecular networks involving oxidative stress, NF-κB, MAPKs, PI3K/Akt, JAK/STAT, NLRP3 inflammasome activation, epithelial barrier dysfunction, immunometabolic reprogramming, ferroptosis, and dysregulated MUC5AC expression. Consequently, therapeutic strategies targeting only a single signaling pathway frequently fail to provide sustained clinical efficacy because of the extensive crosstalk among inflammatory, oxidative, and mucus-regulatory pathways.

This review comprehensively summarizes recent advances in the molecular mechanisms underlying airway inflammation and mucus hypersecretion while highlighting the potential clinical value of natural products derived from marine organisms and terrestrial plants. Marine-derived compounds, including phlorotannins, fucoxanthin, astaxanthin, and fucosterol, together with diverse phytochemicals such as flavonoids, polyphenols, phenolic acids, and triterpenoid saponins, consistently regulate multiple disease-associated signaling pathways. Rather than acting through a single molecular target, these bioactive compounds simultaneously attenuate oxidative stress, suppress inflammatory cytokine production, restore epithelial barrier integrity, inhibit goblet cell differentiation, reduce mucin overproduction, and modulate immunometabolic and ferroptotic responses, thereby offering a multitarget approach for respiratory diseases.

Recent technological advances, including nanoformulation, pulmonary drug delivery, multi-omics, artificial intelligence-assisted drug discovery, and network pharmacology, provide new opportunities to overcome the long-standing limitations associated with natural product development. Nevertheless, successful clinical translation still requires standardized manufacturing processes, rigorous pharmacokinetic evaluation, high-quality randomized clinical trials, and internationally harmonized quality-control systems. Although accumulating preclinical evidence supports the potential of natural products for managing chronic airway diseases, robust clinical evidence remains limited, and routine clinical application cannot yet be recommended. Future interdisciplinary collaboration integrating respiratory biology, pharmaceutical sciences, nutrition, computational biology, and clinical medicine will accelerate the development of evidence-based functional foods, nutraceuticals, and novel respiratory therapeutics. Such integrated approaches are expected to establish natural products as important components of precision respiratory medicine for the prevention and management of chronic airway diseases.

## Figures and Tables

**Table 1:** Representative natural products targeting airway inflammation and mucus hypersecretion.

Natural Product	Source	Experimental Model	Major Molecular Targets	Main Biological Effects	Level of Evidence	Clinical Readiness	Ref.
Dieckol	*Ecklonia cava*	In vitro (A549, RAW264.7 cells); In vivo (PM-induced mouse model)	NF-κB, MAPKs, Nrf2, NLRP3	↓ TNF-α, IL-1β, IL-6, COX-2, iNOS, oxidative stress	In vitro + Animal	Preclinical (In vitro + In vivo)	[3,83,84]
Eckol	*Ecklonia cava*	In vivo (PM-induced airway inflammation model)	NF-κB, MAPKs	↓ Inflammatory cytokines, ↓ epithelial injury	Animal	Preclinical (In vivo)	[84,85,86]
Phlorofucofuroeckol A	*Ecklonia cava*	In vitro (PM-stimulated macrophages)	NF-κB, MAPKs	↓ IL-1β, TNF-α, chemokines	In vitro	Preclinical (In vitro)	[85]
Fucoxanthin	Brown algae	In vitro (BEAS-2B cells); In vivo (OVA-induced asthma mice)	NF-κB, PI3K/Akt, MAPKs, Nrf2	↓ ROS, ↓ goblet-cell hyperplasia, ↓ MUC5AC	In vitro + Animal	Preclinical (In vitro + In vivo)	[87]
Astaxanthin	*Haematococcus pluvialis*	In vivo (PM2.5-, COPD-, and LPS-induced animal models)	AKT, NF-κB, Nrf2/HO-1	↓ Airway inflammation, ↓ oxidative injury, ↓ remodeling	Animal	Preclinical (In vivo)	[88,89,90,91]
Fucosterol	Brown algae	In vitro (cell models); In vivo (animal models)	NF-κB, MAPKs, Nrf2	↓ Pro-inflammatory mediators, antioxidant activity	In vitro + Animal	Preclinical (In vitro + In vivo)	[83,84]
Omega-3 PUFAs and specialized pro-resolving mediators (EPA/DHA, resolvins, protectins, maresins)	Marine fish, microalgae	In vivo (preclinical asthma and COPD animal models)	COX-2-derived lipid mediator pathways, NF-κB	↓ Airway inflammation, ↓ oxidative stress, promotion of inflammation resolution	Animal	Preclinical (In vivo)	[92,93,94,95]
Taxifolin	*Larix* spp.	In vitro (NCI-H292 cells)	NF-κB, (IκBα/p65)	↓ MUC5AC transcription	In vitro	Preclinical (In vitro)	[96]
Quercetin	Fruits, onions	In vivo (cigarette smoke-induced rat model)	EGFR, NF-κB	↓ Airway inflammation, ↓ mucus production	Animal	Preclinical (In vivo)	[97]
Luteolin	Perilla, celery	In vitro (NCI-H292 cells)	NF-κB	↓ MUC5AC expression and secretion	In vitro	Preclinical (In vitro)	[98]
Eriodictyol	*Citrus* spp.	In vitro (NCI-H292 cells)	NF-κB	↓ MUC5AC production	In vitro	Preclinical (In vitro)	[99]
Resveratrol	Grapes	In vitro (human bronchial epithelial cells); In vivo (mouse model)	MAPKs, Nrf2	↓ LPS-induced MUC5AC, ↓ inflammation	In vitro + Animal	Preclinical (In vitro + In vivo)	[100]
Neochlorogenic acid	Plants	In vivo (allergic asthma mouse model)	HO-1, Nrf2	↓ Type-2 inflammation	Animal	Preclinical (In vivo)	[101]
Emodin	*Rheum* spp.	In vitro (NCI-H292 cells)	EGFR–MAPK–Sp1	↓ MUC5AC transcription	In vitro	Preclinical (In vitro)	[102]
Hederacoside C	*Hedera helix*	In vitro (NCI-H292 cells)	MAPKs	↓ EGF-induced MUC5AC	In vitro	Preclinical (In vitro)	[103]
Iristectorigenin A	*Belamcanda chinensis*	In vivo (OVA-induced asthma mouse model)	FOXA3, NOTCH2	↓ Goblet-cell hyperplasia, ↓ mucus secretion	Animal	Preclinical (In vivo)	[104]
Raphanus sativus extract	Radish root	In vitro (NCI-H292 cells)	MUC5AC pathway	↓ MUC5AC gene expression	In vitro	Preclinical (In vitro)	[105]
Vitamin E	Plant oils, nuts, seeds	In vivo (cigarette smoke-induced COPD and experimental asthma models)	NF-κB, Nrf2	↓ Oxidative stress, ↓ airway inflammation, ↓ Th2 cytokines	Animal	Preclinical (In vivo)	[106,108]
Vitamin D	Dietary sources	In vivo (experimental asthma models)	NF-κB, MAPKs, JAK/STAT	↓ Th2 inflammation, ↓ goblet cell hyperplasia, ↓ MUC5AC	Animal	Preclinical (In vivo)	[109]

↓ decrease.

## Data Availability

No new data were created or analyzed in this study. Data sharing is not applicable to this article.

## Abbreviations

Akt	protein kinase B
AP-1	activator protein-1
AREs	antioxidant response elements
CF	cystic fibrosis
DHA	docosahexaenoic acid
COX-2	chronic obstructive pulmonary disease
COPD	chronic respiratory diseases
CRDs	chronic respiratory diseases
DAMPs	damage-associated molecular patterns
EGFR	epidermal growth factor receptor
EPA	eicosapentaenoic acid
ERK	extracellular signal-regulated kinase
Fe^2+^	ferrous iron
FOXA2	forkhead box A2
GCLC	glutamate-cysteine ligase catalytic subunit
GMP	good manufacturing practice
GPX4	glutathione peroxidase 4
GSH	glutathione
HDAC	histone deacetylase
HIF-1α	hypoxia-inducible factor-1 α
HO-1	heme oxygenase-1
IKK	IκB kinase
IL	interleukin
IL-1R	interleukin-1 receptor
ILC2s	group 2 innate lymphoid cells
iNOS	inducible nitric oxide synthase
JAK	Janus kinase
JNK	c-Jun N-terminal kinase
Keap1	Kelch-like ECH-associated protein 1
LDHA	lactate dehydrogenase A
MAPKs	mitogen-activated protein kinases
MUC5AC	mucin 5AC
MUC5B	mucin 5B
NFAT	nuclear factor of activated T cells
NF-κB	nuclear factor-kappa B
NLRP3	NOD-like receptor family pyrin domain-containing 3
NQO1	NAD(P)H quinone oxidoreductase-1
Nrf2	nuclear factor erythroid 2-related factor 2
PDE	phosphodiesterase
PD	pharmacodynamic
PFF-A	phlorofucofuroeckol A
PI3K	phosphatidylinositol 3-kinase
PK	pharmacokinetic
PLGA	polylactide-co-glycolide
PM_2.5_	fine particulate matter
PUFAs	polyunsaturated fatty acids
ROS	reactive oxygen species
SOD	superoxide dismutase
SNARE	SAM-pointed domain-containing ETS transcription factor
SPDEF	SAM-pointed domain-containing ETS transcription factor
SPMs	specialized pro-resolving mediators
STAT	signal transducer and activator of transcription
STING	stimulator of interferon genes
TAK1	transforming growth factor-β-activated kinase 1
TGF	transforming growth factor
T2	type 2
TLRs	toll-like receptors
TMEM16A	TMEM16A, transmembrane member 16A
TNF-α	tumor necrosis factor-alpha
TNFR	tumor necrosis factor receptor
TRP	transient receptor potential
TRPA1	transient receptor potential ankyrin 1
TRPV1	transient receptor potential vanilloid 1
TSLP	thymic stromal lymphopoietin
